# Extracellular vesicle characteristics and microRNA content in cerebral palsy and typically developed individuals at rest and in response to aerobic exercise

**DOI:** 10.3389/fphys.2022.1072040

**Published:** 2022-12-21

**Authors:** Ivan J. Vechetti, Jessica Norrbom, Björn Alkner, Emma Hjalmarsson, Alexandra Palmcrantz, Eva Pontén, Jessica Pingel, Ferdinand von Walden, Rodrigo Fernandez-Gonzalo

**Affiliations:** ^1^ Department of Nutrition and Health Sciences, College of Education and Human Sciences, University of Nebraska-Lincoln, Lincoln, NE, United States; ^2^ Department of Physiology and Pharmacology, Karolinska Institutet, Stockholm, Sweden; ^3^ Department of Orthopaedics, Eksjö, Region Jönköping County and Department of Biomedical and Clinical Sciences, Linköping University, Linköping, Sweden; ^4^ Division of Pediatric Neurology, Department of Women’s and Children’s Health, Karolinska Institutet, Stockholm, Sweden; ^5^ Department of Pediatric Orthopedic Surgery, Karolinska University Hospital, Stockholm, Sweden; ^6^ Department of Neuroscience, University of Copenhagen, Copenhagen, Denmark; ^7^ Division of Clinical Physiology, Department of Laboratory Medicine, Karolinska Institutet, Stockholm, Sweden; ^8^ Unit of Clinical Physiology, Karolinska University Hospital, Stockholm, Sweden

**Keywords:** exosomes, endurance exercise, miR-486, skeletal muscle, frame running

## Abstract

In this study, the properties of circulating extracellular vesicles (EVs) were examined in cerebral palsy (CP) and typically developed (TD) individuals at rest and after aerobic exercise, focusing on the size, concentration, and microRNA cargo of EVs. Nine adult individuals with CP performed a single exercise bout consisting of 45 min of Frame Running, and TD participants completed either 45 min of cycling (*n* = 10; TD EX) or were enrolled as controls with no exercise (*n* = 10; TD CON). Blood was drawn before and 30 min after exercise and analyzed for EV concentration, size, and microRNA content. The size of EVs was similar in CP vs. TD, and exercise had no effect. Individuals with CP had an overall lower concentration (∼25%, *p* < 0.05) of EVs. At baseline, let-7a, let-7b and let-7e were downregulated in individuals with CP compared to TD (*p* < 0.05), while miR-100 expression was higher, and miR-877 and miR-4433 lower in CP compared to TD after exercise (*p* < 0.05). Interestingly, miR-486 was upregulated ∼2-fold in the EVs of CP vs. TD both at baseline and after exercise. We then performed an *in silico* analysis of miR-486 targets and identified the satellite cell stemness factor Pax7 as a target of miR-486. C2C12 myoblasts were cultured with a miR-486 *mimetic* and RNA-sequencing was performed. Gene enrichment analysis revealed that several genes involved in sarcomerogenesis and extracellular matrix (ECM) were downregulated. Our data suggest that circulating miR-486 transported by EVs is elevated in individuals with CP and that miR-486 alters the transcriptome of myoblasts affecting both ECM- and sarcomerogenesis-related genes, providing a link to the skeletal muscle alterations observed in individuals with CP.

## Introduction

Extracellular vesicles (EVs) are a heterogeneous family of particles encompassed by a membrane that originates from the plasma membrane or endosome. Once believed to “just” serve as a disposal mechanism (Pan and Johnstone, 1983), EVs are now highlighted as an important form of intercellular communication with multiple roles in both physiological and pathophysiological processes (Abels and Breakefield, 2016). Indeed, EVs are capable of delivering molecular cargo such as mRNAs, microRNAs, proteins, and lipids to recipient cells (Février and Raposo, 2004; Raposo and Stoorvogel, 2013), with the potential to impact multiple cellular processes.

In response to exercise, skeletal muscle secretes molecules, including EVs, that mediate or even directly cause adaptations to non-skeletal muscle tissue, which in turn influences exercise adaptations (Estébanez et al., 2021). Skeletal muscle fibers secrete EVs with different functions affecting liver processes (Whitham et al., 2018) or promoting lipolysis in white adipose tissue (Vechetti et al., 2021a), to name a few examples. The actions triggered by exercise-induced EVs are suggested to be due to transient alterations in vesicular cargo and increased EV number, without modifications in vesicle size (Estébanez et al., 2021). For example, the concentration of miR-1 in circulating EVs increases in response to aerobic exercise and return to baseline concentrations 4 h after the exercise bout (D’Souza et al., 2018). Other skeletal muscle-enriched microRNAs in EVs that have been described to respond to acute exercise are miR-133a, miR-133b, miR-206, miR-208a, and miR-499 (Yin et al., 2019), highlighting exercise as an acute modifier of skeletal muscle EV content.

Cerebral palsy (CP) is a neurological disorder caused by a non-progressive brain injury. CP is the most common cause of motor impairment in children (Oskoui et al., 2013). After the initial insult, skeletal muscle function typically deteriorates over time (Hägglund and Wagner, 2011). Skeletal muscles of individuals with CP are smaller, weaker, and differ with respect to composition as compared with muscles from typically developed (TD) individuals. Specifically, increased collagen (Booth et al., 2001) and intramuscular adipose tissue content (Noble et al., 2014) is believed to contribute to poor muscle function. Individuals with CP respond to exercise, although differences in performance and adaptations exist when compared with TD individuals (Hjalmarsson et al., 2020; von Walden et al., 2020). Extracellular vesicle biology has never been investigated in individuals with CP, and it remains to be shown if the response of circulating EVs induced by exercise mimics that of TD individuals. In addition, the microRNA profile of individuals with CP has only been studied in neonates at risk for CP (Chapman et al., 2018). Data related to circulating microRNAs in CP adults, with the consequent phenotypic and molecular alterations induced by the long-term effects of CP, is non-existent.

With this background, we performed a study to (i) investigate potential differences in circulating EVs in terms of concentration and size between individuals with CP and controls (i.e., TD), and (ii) examine the response of microRNA cargo in EVs of CP and TD individuals after an acute bout of aerobic exercise. We hypothesized that there would be differences in the EV concentration at baseline between CP and TD individuals, and that specific microRNAs would be differentially expressed after exercise between these two groups.

## Materials and methods

### Study design

Nine individuals with CP performed a 45-min Frame Running (previously called RaceRunning) exercise bout (CP EX). Physically active TD individuals completed either 45 min of continuous cycling on a cycle ergometer (10 participants; TD EX) or were enrolled as controls with no exercise intervention (10 participants; TD CON). During the exercise bouts, heart rate and perceived level of exertion were registered. Blood samples were obtained before and 30 min post exercise. Plasma was obtained and used for analysis of EV concentration, size, and microRNA content. *In-silico* and *in-vitro* analyses were then performed to confirm the human data.

### Participants

Individuals with CP from different Frame Running associations in Stockholm greater area volunteered for this study. TD participants were recreationally active individuals, i.e., involved in aerobic exercise (AE) one to three times per week and/or resistance exercise once or twice per week. Inclusion criteria were 18–50 years of age for the TD individuals and 16–50 years of age for the CP group. Exclusion criteria for the TD individuals were cardiovascular disease, neuromuscular disease, or severe knee problems, and for the CP group it was inability to participate in Frame Running due to motor difficulties and/or failure to understand and follow instructions. Thus, the CP group consisted of five male and four female participants [age; 27 years (min-max; 16-44), height; 163 cm (146–175 cm), weight; 53 kg (34–67 kg), BMI; 19.7 (14.7-24.6)], with a Gross Motor Function Classification System (GMFCS, scale from 1 to 5) between 2 and 4 (median GMFCS 3). Three of them had spastic CP, five had dyskinetic CP, and one had ataxic CP. The TD EX group consisted of six male and four female participants [age; 28 years (18-41), height; 176 cm (159–193 cm), weight; 79 kg (46–110 kg), BMI; 25.2 (18.4-34)]. The TD CON group consisted of six male and four female participants [age; 31 years (19-50), height; 178 cm (165–193 cm), weight; 81 kg (57–102 kg), BMI; 25.5 (20.7-32.0)]. All participants gave their informed written consent to participate, after receiving written and oral information about the study. In addition, a legal guardian provided written informed consent to participate for those individuals under 18. The study protocols were approved by the Regional Ethical Review boards in Linköping (#2017/183-31) and Stockholm (#2016/1139-31/2 with addendums 2016/1675-32, 2017/2237-32, 2019-01008).

### Exercise bouts and blood sampling

All participants were asked to refrain from exercise 48 h before any activity included in this study. The exercise bout for TD EX consisted of 45 min of cycling (Monark 828 E, Monark Exercise AB, Vansbro, Sweden) at 70% of estimated VO_2max_, which was assessed a week prior to the acute bout using the Ekblom-Bak test (Björkman et al., 2016). Participants rated their own perceived level of exertion every 10 min using the Borg’s RPE scale and their heart rate was monitored continuously (Garmin Edge 25, Garmin, United States).

The exercise bout for CP EX consisted of 45 min of Frame Running (Hjalmarsson et al., 2020). Heart rate and perceived level of exertion was monitored following the same protocol as described for the TD EX group. Frame Running is a parasport adapted for people with movement disability. It consists of a custom-built tricycle with a saddle and a chest plate but no pedals, which provides the individual balance and stability for locomotion.

Blood was obtained at rest before the exercise bout and at 30 min post exercise *via* a peripheral venous catheter in individuals from TD EX and CP EX. For participants in TD CON, blood was sampled at rest at two occasions separated by 75 min. Plasma (1 ml) was prepared as previously described (von Walden et al., 2021) and kept at −80°C until further analysis.

### Extracellular vesicle isolation from human plasma

Prior to EVs isolation, plasma was centrifuged twice at 3000 × g for 20 min. The resulting supernatant was then filtered through a 0.22 µm syringe-driven filter to remove large contaminating vesicles. Following the filtration, EVs were isolated using ExoEasy membrane affinity column (Enderle et al., 2015) according to the manufacturer’s directions (Qiagen, Carlsbad, CA, United States). Briefly, filtered plasma was mixed 1:1 with 2x binding buffer and then added to the membrane affinity column. After centrifugation, the column was washed, and extracellular vesicles eluted in 400 µL of elution buffer. As a quality control, EVs were visualized using transmission electron microscopy as previously described (Vechetti et al., 2021a) (Supplementary Figure S1).

### Nanoparticle tracking analysis

Extracellular vesicle size and concentration were measured by nanoparticle tracking analysis (NTA) using a ZetaView^®^ (Particle Metrix, Meerbusch, Germany) instrument. Isolated EV samples were appropriately diluted using 1X DPBS buffer (Life Technologies, Carlsbad, CA, United States) to measure the particle size and concentration. All NTA measurements were recorded and analyzed at 11 positions. The ZetaView system was calibrated using 100 nm polystyrene particles. Temperature was maintained around 25°C. Prior to each analysis, camera sensitivity and focus were adjusted to ensure the sensitivity remained constant throughout the analysis.

### RNA isolation and small RNA sequencing

Total RNA was isolated from EVs using Qiazol Reagent (Qiagen, Carlsbad, CA, United States) and ExoRNAeasy kit (Qiagen) according to the manufacturer’s instructions. The RNA yield and size distribution were analyzed using an Agilent 2.100 Bioanalyzer with an RNA 6,000 Pico kit (Agilent Technologies, Foster City, CA, United States). Samples were shipped on dry ice for small RNA sequencing, performed by Norgen Biotek (Thorold, Ontario, Canada). For small RNA sequencing, pre- and post-exercise samples were pooled to generate an *N* = 3 per timepoint per group (TD EX; two groups of *n* = 3, i.e., 6 men, and one group of *n* = 4, i.e., 4 women, CP EX; three groups of *n* = 3 grouped by GMFCS levels–II, III, and IV). The complete data set is publicly available at Gene Expression Omnibus with accession ID (GSE219100).

### In silico prediction of miR-486 target genes

To identify the possible targets of miR-486 we performed *in silico* prediction as described before (Valentino et al., 2021). Briefly, we used miRanda (Enright et al., 2003) and RNAhybrid (Krüger and Rehmsmeier, 2006) software for target prediction and utilized a custom Python script to select only shared miRNA:target gene between the two software programs with a minimal free energy set as ΔG° = −18 kJ.

### C2C12 culture and miR-486 mimetic

The skeletal muscle C2C12 (ATCC, CRL-1772) myoblasts were cultured in Dulbecco’s Modified Eagles Medium (DMEM) supplemented with 10% Fetal Bovine Serum (FBS), 100 units/ml of Penicillin and 100 μg/ml of Streptomycin until reaching 60%–70% confluency. C2C12 myoblasts were transfected with either a mirVana miR-486 mimic or a scramble miRNA sequence (Life Technologies) used as a negative control. This control sequence does not target any known mature miRNA. Transfection was performed using lipofectamine 2,000 (Life Technologies), according to the manufacturer’s instructions. Shortly, lipofectamine complexes were stabilized in OptiMEM (Gibco) for 5 min and incubated with the miRNA probes, diluted to 25 nM in OptiMEM, for 20 min. Finally, the lipofectamine/probe complexes were added to the cells in a medium without antibiotics. After 6 h of incubation, fresh media was added, and cells were harvested following 24 h. Each transfection experiment was independently repeated at least in triplicate. RNA extraction was performed using TRI reagent (Sigma-Aldrich, St. Louis, MO, United States) coupled with further columns-based processing using the Direct-zol Kit (Zymo Research, Irvine, CA, United States). RNA sequencing was performed by Novogene on a Illumina HiSeq using 150 bp paired-end sequencing (Murach et al., 2022). The complete data set is publicly available at Gene Expression Omnibus with accession ID (GSE219100).

### Data analysis

Heart rate and Borg’s scale data was compared between TD EX and CP EX employing a two-tailed unpaired *t*-test. Potential differences in EV concentration and size across groups and in response to exercise were investigated using a mixed model analysis with group (CP EX, TD EX, TD CON) and time (Pre, 30 min Post) as factors. If a significant interaction was seen, Bonferroni *post hoc* corrections were performed. The significance level was set at 5% (*p* < 0.05). Statistical analyses were performed using Prism 7 for Mac OS X (GraphPad Software, San Diego, CA) and Jamovi (v1.6.23.0).

For the microRNA-seq analysis, library preparation and sequencing were performed by Norgen Biotek using the Small RNA Library Prep Kit (Cat. 63600). Sequencing was performed using the NextSeq 500/550 High Output Kit v2 (51 Cycles using a 75-Cycle Kit) on the Illumina NextSeq 500 platform. Single-end reads for miRNAs were mapped to miRbase version 22 using BowTie2. The alignment was performed by Norgen Biotek using sRNAbench and sRNAtoolbox. Raw counts were acquired with featurecounts and they were used as input for the statistical analysis, using DESeq2 (Love et al., 2015) and other Bioconductor packages in R3.6.2. MicroRNAs with less than 10 counts were removed from the analysis. A model taking into account the genotype (TD or CP) and activity (EX or CON) was built and the results were considered statistically significant at an adjusted *p* < 0.05 (FDR: Benjamini–Hochberg). DESeq2’s regularized-logarithm transformation (rlog) of the count data was used to generate a matrix of regularized counts for sample visualizations.

For target prediction analysis, we utilized the common targets from two different prediction software as previously described by us (Valentino et al., 2021). Briefly, target genes were detected using miRanda (Enright et al., 2003) and RNAhybrid (Krüger and Rehmsmeier, 2006) software, followed by a custom Python script to select only shared miRNA:target gene between the two software programs with a minimal free energy set as ΔG° = −18 kJ. To determine the strength of the predicted miR-486 to pax7 target pairing, we evaluated seed region match quality using the RNAhybrid program (Krüger and Rehmsmeier, 2006) to calculate the minimal free energy of the hybridization.

Gene ontology (GO, sources; Reactome, Wikipathways, BioCarta) and Kyoto Encyclopedia of Genes and Genomes (KEGG) pathway enrichment analyses were carried out in cluster profiler (Yu et al., 2012) on the predicted target genes. The GO terms and KEGG pathways with FDR <0.05 were considered to be significantly enriched.

## Results

Data originating from the same participants (TD) has been previously used to investigate the effects of acute exercise on mitochondria-derived peptides in healthy participants (von Walden et al., 2021).

### Heart rate and rate of perceived exertion

Average heart rate during the exercise bout was similar (*p* > 0.05) between CP EX (150 ± 32 bpm) and TD EX (157 ± 18 bpm). Likewise, the rate of perceived exertion averaged over the exercise bout was not different (*p* > 0.05) between CP EX (13.9 ± 1.6) and TD EX (14.6 ± 0.9).

### EV concentration and size

**FIGURE 1:**
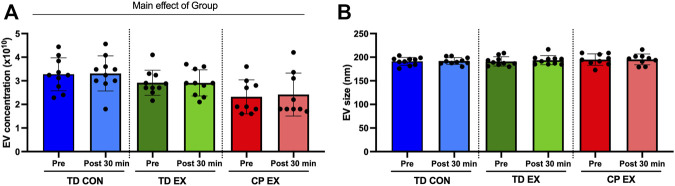
Extracellular vesicle (EV) characterization in typically developed (TD) and cerebral palsy (CP) individuals and its response to exercise. **(A)**; EV concentration (particles per mL of plasma) before (Pre) and after (Post) 30 min of aerobic exercise (EX) or control (CON). **(B)**; EV size before (Pre) and after (Post) 30 min of EX or CON condition.

There was a significant main effect of group (*p* = 0.003, F = 7.1) in EV concentration in plasma (Figure 1A). This effect was mainly driven by a lower concentration of EVs in individuals with CP compared with TD participants. Exercise did not affect EV concentration in any of the groups (Figure 1A). No differences were found in EV size across groups (Figure 1B). Also, exercise did not have any effect on EV size (Figure 1B).

### microRNA cargo in EVs

**FIGURE 2:**
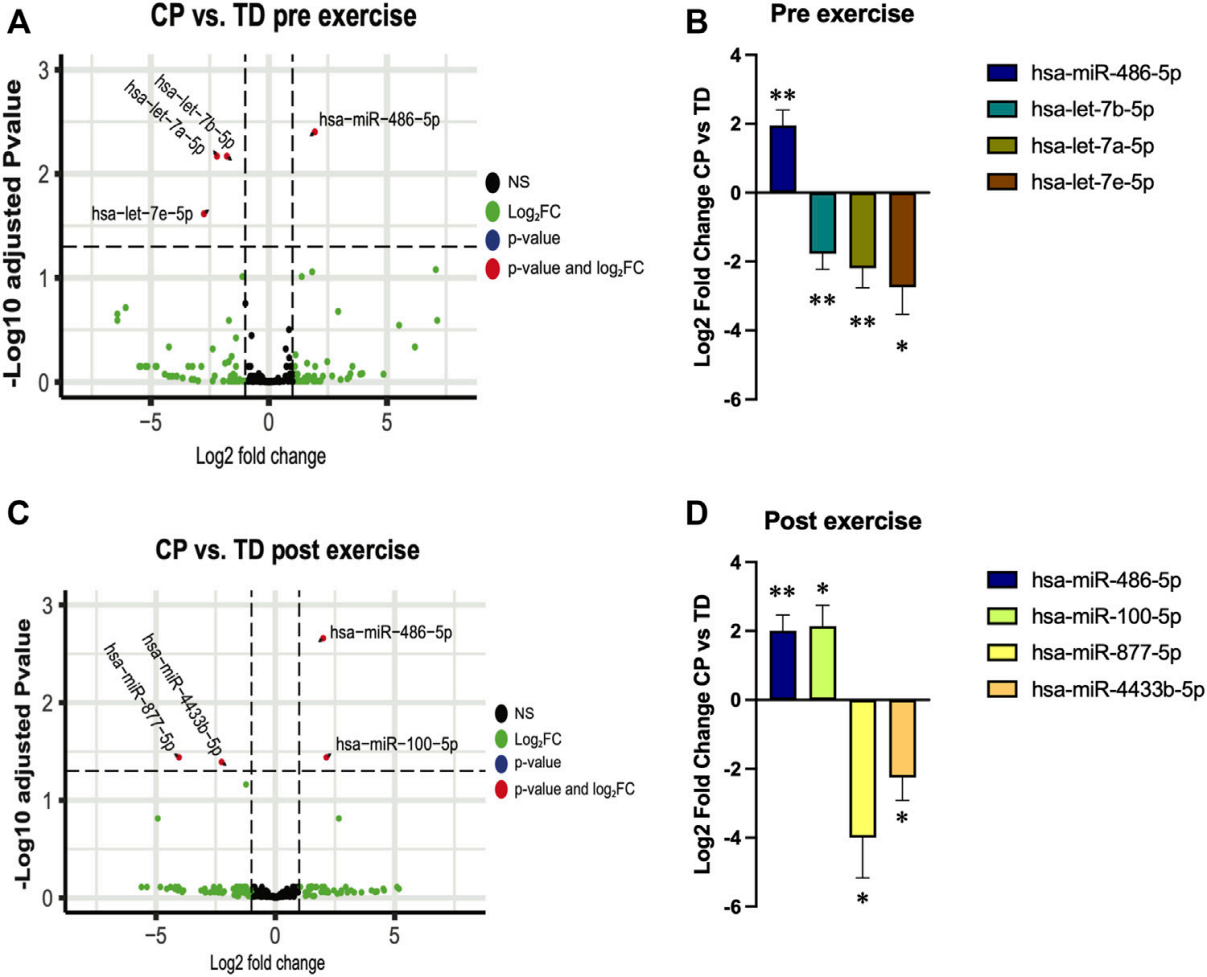
Extracellular vesicles microRNA cargo differences between cerebral palsy (CP) and typically developed (TD) individuals before (pre exercise) and 30 min after (post exercise) a bout of aerobic exercise. **(A,C)**; volcano plots illustrating changes in EV cargo between TD and CP before **(A)** and after **(C)** exercise. Each point on the scatter plot represents a microRNA where its position on the *x*-axis corresponds to the average log2-fold change after exercise and the position on the *y*-axis corresponds to log10 *p*-value of its differential expression after adjustment for false discovery rate. **(B,D)**; bar graphs illustrating the expression of selected microRNAs in CP individuals relative to TD participants before **(B)** and after **(D)** exercise; **; *p* < 0.01, *; *p* < 0.05.

At baseline (rest), miR-486 was higher, and let-7a, let-7b, and let-7e lower in individuals with CP when compared with TD participants (Figures 2A, B; Supplementary Table S1). Thirty minutes after exercise miR-486 remained upregulated in CP vs. TD. In addition, miR-100 expression was higher, and miR-877 and miR-4433 lower in CP EX than in TD EX (Figures 2C, D; Supplementary Table S1). Within-group analysis of sequencing data is shown in Supp. Table 1. Exercise did not affect EV cargo in any of the groups (Supplementary Figure S2).

**FIGURE 3:**
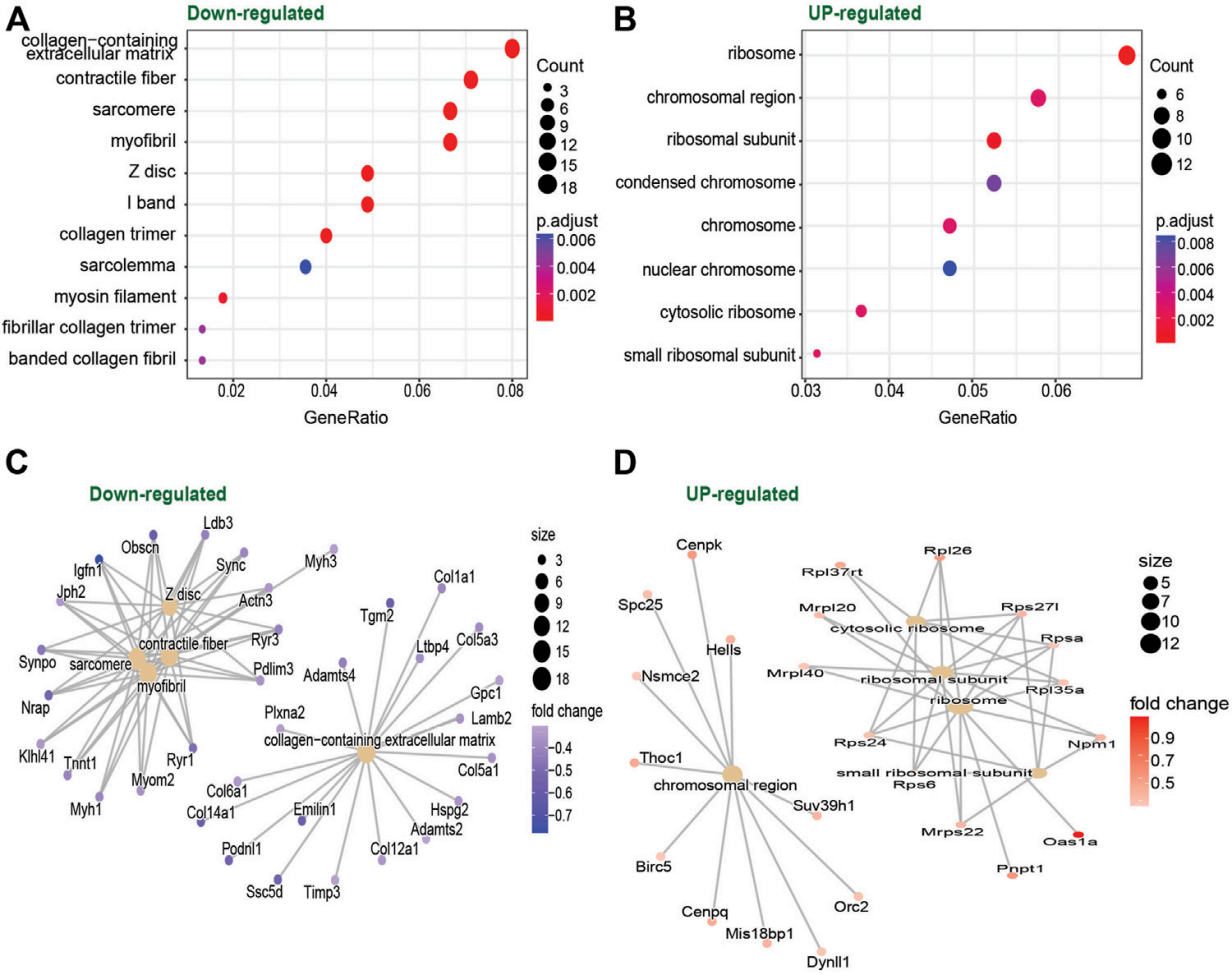
Dot plot of gene ontology enrichment of Down-regulated **(A)** and Upregulated **(B)** genes in C2C12 cells culture with vs. without miR-486. Red indicates higher enrichment; blue indicates lower enrichment. The sizes of the dots represent the gene counts of each row (GO category). Enrichment map of the inter-relation of the top 5 enriched biological processes on the Downregulated **(C)** and Upregulated **(D)** genes.

With these results at hand, we decided to focus our analysis on the muscle-specific miR-486, which was ∼2-fold upregulated in TD at both baseline and after exercise. Our prediction model (Supplementary Figure S3) indicated that, among other genes, miR-486 targets Pax7, an important gene for skeletal muscle progenitor cell (SMPC) stemness, supporting previous reports (Dey et al., 2011). To further characterize the impact of miR-486 on the SMPC transcriptome, we cultured C2C12 cells with a miR-486 mimetic. Our results showed that genes related to sarcomere, contractile fiber, and myofibril, as well as genes related to extracellular matrix (ECM), were downregulated by the miR-486 mimetic (Figures 3A, C). In contrast, genes involved in protein translation (ribosome) seemed to be upregulated (Figures 3B, D). The complete list of differentially expressed genes and gene ontologies is presented in Supplementary Table S2. To deepen our understanding and interpretation of the changes induced by the miR-486 mimetic, we compared the differentially expressed gene list obtained from the current C2C12 cell data set with DEG lists originated from hamstrings (Smith et al., 2012) and wrist (Smith et al., 2009) skeletal muscle of CP individuals, as well as muscle from miR-486-KO mice (Samani et al., 2022) (Supplementary Figure S4 and Supplementary Table S2). Among the genes differentially expressed in our C2C12 experiments and in CP skeletal muscle, we identified mediators of insulin growth factors (*IGFBP5*), regulators of the organization of the actin filament system (*RHOBTB1*), several collagen related genes (e.g., *COL1A1, COL3A1, COL4A1, COL5A1*), and myosin heavy chain (*MYH1, MYH3*) related genes. Four common genes were identified when cross-referencing the CP hamstring, miR-486-KO and C2C12 data sets related either to the ECM (*COL1A1, COL1A2)* or the cytoskeleton (*MAP1A, PLXNA2*).

## Discussion

In this study, the characteristics of circulating EVs were compared between CP and TD individuals at rest and after acute aerobic exercise. Our results indicate that the size of plasma EVs is similar between CP and TD and that exercise does not affect this morphological characteristic. However, CP individuals presented an overall lower concentration of EVs both at baseline and after exercise. The microRNA cargo transported by circulating EVs was different between the CP and TD groups, and exercise influenced these differences. Interestingly, the skeletal muscle specific miRNA miR-486 was upregulated in EVs from CP individuals both at baseline and after exercise. Our *in silico* and *in-vitro* follow-up experiments showed that miR-486 targets Pax7, an important regulator of satellite cells, and that it affects the transcriptome of skeletal muscle progenitor cells related to sarcomerogenesis and ECM.

The effect of exercise on EV concentration is a matter of current debate (Estébanez et al., 2021). Our results support the studies indicating that acute exercise does not affect EV concentration (Lovett et al., 2018; Brahmer et al., 2019; Rigamonti et al., 2020). On the other hand, there seems to be agreement that EV size remains constant after exercise (Estébanez et al., 2021), a notion supported by the current results. An interesting finding of this study was that individuals with CP appeared to have a lower concentration of EVs compared to TD individuals. Lower EV concentration has been previously described in elderly *versus* young individuals (Eitan et al., 2017) and could indicate suboptimal intercellular and consequently interorgan communication. It remains to be shown what cell type(s) are responsible for this lower EV release in individuals with CP.

The molecular cargo transported and delivered by EVs has a major impact on cell-to-cell communication, as well as on the crosstalk between different organs. A novel finding of our analysis was that EVs from individuals with CP had significantly lower expression of three microRNAs, namely let-7a, let-7b, and let-7e, at baseline compared with TD individuals. Reduced levels of let-7 family microRNAs in circulating EVs have been linked to increased fibrosis in the liver (Matsuura et al., 2016). One could speculate that the low let-7 content in EVs reported in the current study could be associated with the well-known skeletal muscle phenotype of CP individuals showing a marked increased collagen content (Booth et al., 2001; Bruin et al., 2014; Von Walden et al., 2018). Another important function of let-7 microRNAs is the regulation of skeletal development, with let-7 deficiencies leading to an impairment in skeletal growth (Papaioannou et al., 2013). With this background, it could be that the lower let-7 levels found in participants with CP in this investigation could explain, at least partly, the generally shorter stature compared to TD individuals (Ruiz Brunner et al., 2022). Even though the two hypotheses linking reduced levels of circulating let-7 with muscle ECM changes and reduced height in individuals with CP need to be specifically tested in future studies, we show that aerobic exercise normalized (i.e., similar to TD individuals) circulating let-7 levels, which could be interpreted as an exercise-induced adaptation towards a healthier EV-content profile.

Exercise is a powerful stimulus to influence the content of EVs present in the bloodstream (Vechetti et al., 2021b). After an aerobic exercise bout, miR-877 and miR-4433b were downregulated and miR-100 was upregulated in CP compared with TD individuals. To our knowledge, this is the first report indicating effects of exercise on miR-877 and miR-4433b expression in EVs. However, circulating miR-100 has been shown to decrease after exercise in trained individuals (Sansoni et al., 2018). Thus, it appears that individuals with CP responded to the exercise performed in a more naïve or untrained fashion compared to TD participants. This is not surprising given that individuals with CP generally engage in lower levels of high-intensity physical activity than their TD peers (Nooijen et al., 2014).

An important and novel finding of the current study was the ∼2-fold elevated levels of miR-486 in EVs from CP individuals compared with TD subjects, both at baseline and after exercise. A possible explanation for such differences may be the lower physical fitness status of CP individuals compared with TD, as previously mentioned, since there are reports indicating that chronic exercise decreases circulating miR-486 levels (Aoi et al., 2013; Barber et al., 2019). The host gene of miR-486 is ankyrin-1, and this gene has been shown to be upregulated in skeletal muscle from CP individuals (Smith et al., 2012). Therefore, it is possible that the differences in circulating levels of miR-486 in the current study had an origin and/or important effect on skeletal muscle tissue. To test this hypothesis, we performed a target prediction model for miR-486, which showed that one of the target genes of this microRNA is Pax-7. Previous reports have indicated that Pax-7 is downregulated by miR-486 (Dey et al., 2011). Pax-7 is an important transcription factor for satellite cell (skeletal muscle progenitor cell) function in adult skeletal muscle (von Maltzahn et al., 2013) and consequently plays a critical role in myogenesis. Pax-7 is expressed in proliferating satellite cells and undergoes rapid downregulation during satellite cell differentiation. The results presented in this study showing higher expression of miR-486 in individuals with CP may therefore indicate a miR-486-induced mismatch between proliferation and differentiation of satellite cells. This mismatch could lead to altered numbers of viable and normally functioning myofibers in skeletal muscle from CP individuals, as previously suggested (Domenighetti et al., 2018). On the other hand, contrasting reports indicate a beneficial effect of miR-486 on muscle health, reversing skeletal muscle defects in an animal model of Duchenne muscular dystrophy (Wang et al., 2022). Thus, an alternative interpretation of the greater miR-486 abundance in EVs from CP individuals could be an attempt, by the muscle itself, to counteract the negative effects of CP on skeletal muscle.

To further investigate the role of miR-486 in skeletal muscle cells, we cultured C2C12 cells (immortalized mouse myoblast cell line) with a miR-486 mimetic. The results showed that several genes involved in sarcomerogenesis and ECM were downregulated. Downregulation of these groups of genes has been previously reported in skeletal muscle disuse atrophy (Urso et al., 2006; Chen et al., 2007). The similarities between acute (days or weeks) disuse atrophy and a chronic condition affecting the skeletal muscle, i.e., CP, cannot be overlooked. Indeed, individuals with CP exhibit weak and thin muscles (von Walden et al., 2017) with altered ECM properties (Von Walden et al., 2018; Smith et al., 2021). Sarcomerogenesis and ECM changes driven at least in part by miR-486 thus appear to be a common signature of muscle deconditioning. When we compared the current C2C12 dataset with other CP- and miR-486 related datasets, we found common genes related to cellular structure (genes coding for myosin and actin), as well as a set of genes coding for different types of collagens. Overall, these data seem to indicate overlapping transcriptional regulation between skeletal muscle in CP individuals and the direct effects of miR-486. However, such a relationship should be investigated in further detail.

In our study, exercise did not alter the expression of any EV-transported microRNA. This is in contrast with other studies showing that acute endurance exercise alters circulating microRNAs, e.g., miR-20a and miR-21 (Zhou et al., 2020). Differences across studies in relation to the source of the microRNAs [i.e., plasma/serum vs. EVs (D’Souza et al., 2018)], the type of exercise (Sieland et al., 2021), the timepoints selected, and the specific population investigated may explain, at least partly, these contrasting findings.

In summary, this study provides novel and valuable information on the properties and cargo of EVs in CP individuals at rest and after aerobic exercise. The size of EVs was similar in all groups and independent of the exercise stimulus. Conversely, CP individuals had a lower concentration of EVs both at baseline and after exercise. TD and CP participants presented a different EV-transported microRNA profile at rest, and exercise affected these differences. However, the upregulation of miR-486 in EVs from CP individuals was stable and evident both at baseline and after exercise. Our *in silico* and *in vitro* follow-up experiments showed that miR-486 targets Pax7, an important regulator of satellite cells, and that miR-486 affects the expression of genes responsible for sarcomerogenesis and ECM in skeletal muscle cells. Overall, our data suggest that miR-486 is stably overrepresented in CP EVs, plausibly providing a link to skeletal muscle alterations seen in individuals with CP.

## Data Availability

The transcriptomic datasets presented in this study can be found in online repositories. The names of the repository/repositories and accession number(s) can be found below: https://www.ncbi.nlm.nih.gov/, GSE219100. Other data generated during the current study are available from the corresponding author on reasonable request.
