# Supplementary material for: Extracellular vesicle characteristics and microRNA content in cerebral palsy and typically developed individuals at rest and in response to aerobic exercise
Source: Front Physiol. 2022 Dec 21;13:1072040. doi: 10.3389/fphys.2022.1072040 (PMC9811128; doi:10.3389/fphys.2022.1072040)
Supplement: Supplementary file 2 [file Image4.pdf]

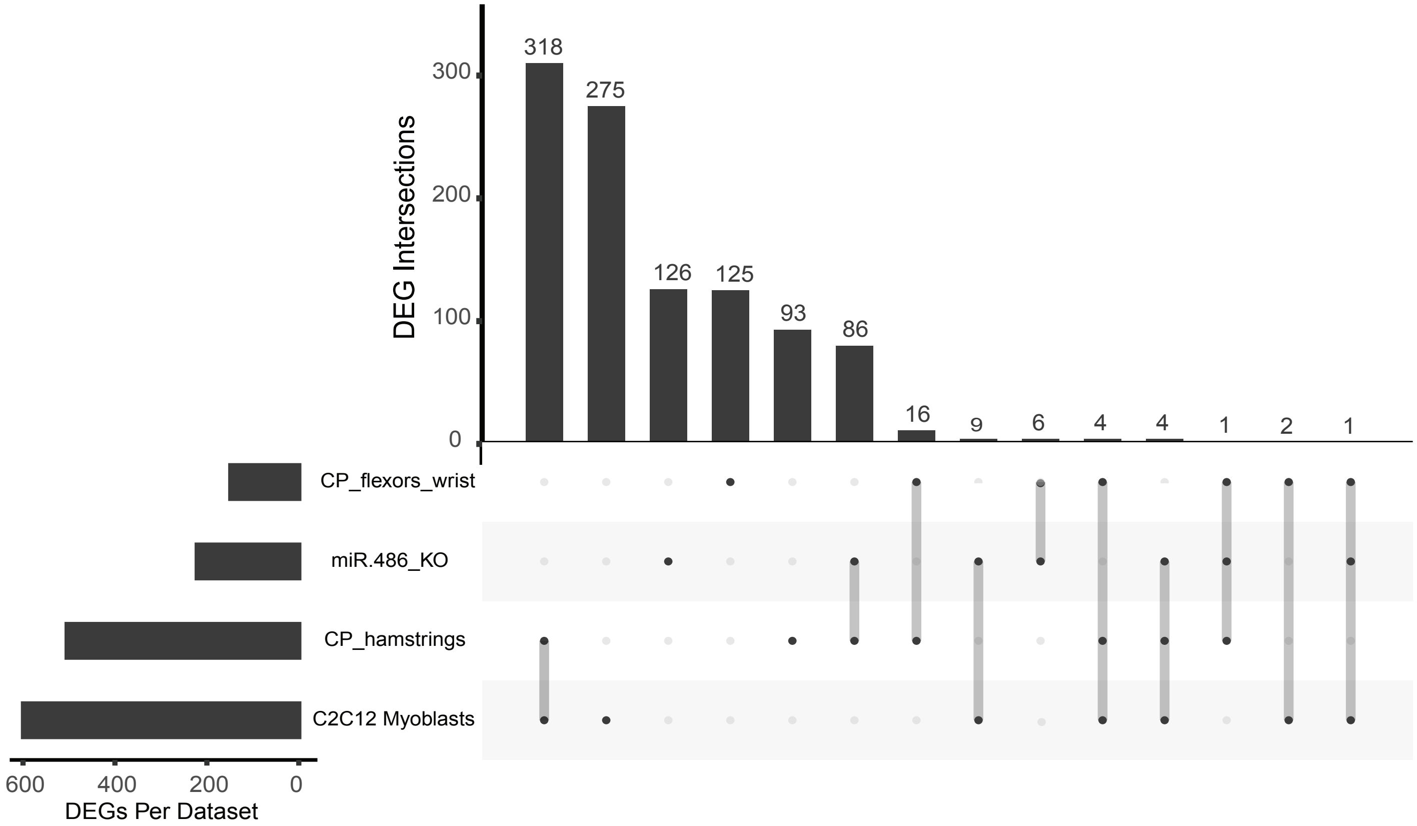

Supplementary Figure 4 - UpSet plot (Lex et al., 2014) representing the number of intersections of differentially expressed genes (DEGs) amongst different datasets (Smith et al., 2012; CP_hamstrings, Smith et al., 2009; CP_flexors_wrist, Samani et al., 2022; miR-486_KO, and current study; C2C12 myoblast treated with miR-486 mimic). Horizontal bars represent the total number of DEG within the datasets. The vertical bars represent the number of DEG in the highlighted comparisons (filled black circles along the x axis). A line linking multiple filled circles represents a column in which those DEGs are shared among more than one group comparison.
