# Supplementary material for: Extracellular vesicle characteristics and microRNA content in cerebral palsy and typically developed individuals at rest and in response to aerobic exercise
Source: Front Physiol. 2022 Dec 21;13:1072040. doi: 10.3389/fphys.2022.1072040 (PMC9811128; doi:10.3389/fphys.2022.1072040)
Supplement: Supplementary file 3 [file Image2.pdf]

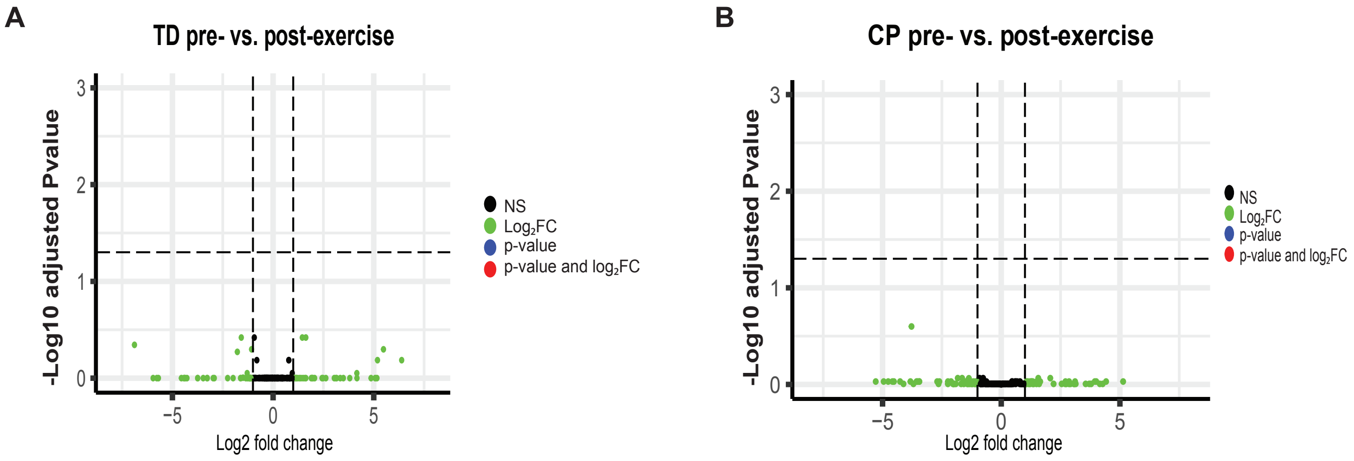


**Supplementary Figure 2** – Volcano plots illustrating differences in EV cargo after exercise in (A) typically developed (TD) and (B) cerebral palsy (CP) individuals. Each point on the scatter plot represents a microRNA where its position on the x-axis corresponds to the average log2-fold change after exercise. The position on the y-axis corresponds to log10 P value of its differential expression after adjustment for false discovery rate.
