# Supplementary material for: Extracellular vesicle characteristics and microRNA content in cerebral palsy and typically developed individuals at rest and in response to aerobic exercise
Source: Front Physiol. 2022 Dec 21;13:1072040. doi: 10.3389/fphys.2022.1072040 (PMC9811128; doi:10.3389/fphys.2022.1072040)
Supplement: Supplementary file 4 [file Image3.pdf]

```
dataset: 1
target: Pax7
length: 6207
miRNA : miR-486
length: 21

mfe: -32.2 kcal/mol
p-value: undefined

position  1058
target 5' A    C      AAGCC         U 3'
           UCGG GGCAGC     CAG ACAGG
           AGCC CCGUCG     GUC UGUCC
miRNA  3'             A       A     U 5'
```

miR-486

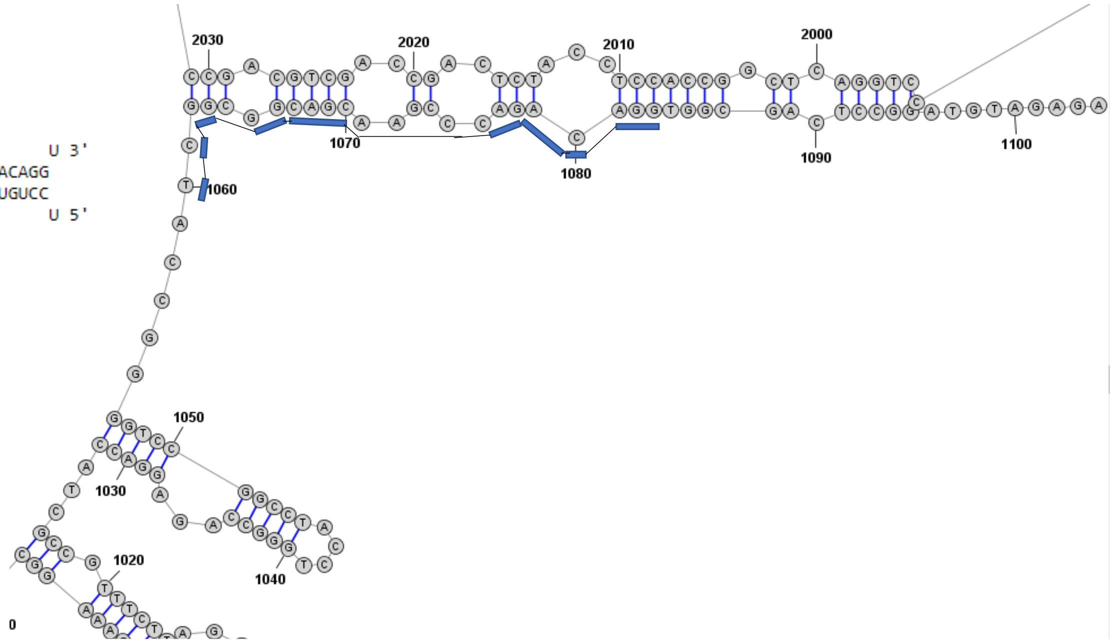

Supplementary Figure 3 – miRNA binding site prediction on the 3’UTR of Pax7. Blue bars represent miR-486 prediction site. Free energy (mfe): -32.2 kcal/mol.
