# Supplementary material for: Extracellular vesicle characteristics and microRNA content in cerebral palsy and typically developed individuals at rest and in response to aerobic exercise
Source: Front Physiol. 2022 Dec 21;13:1072040. doi: 10.3389/fphys.2022.1072040 (PMC9811128; doi:10.3389/fphys.2022.1072040)
Supplement: Supplementary file 6 [file Image1.pdf]

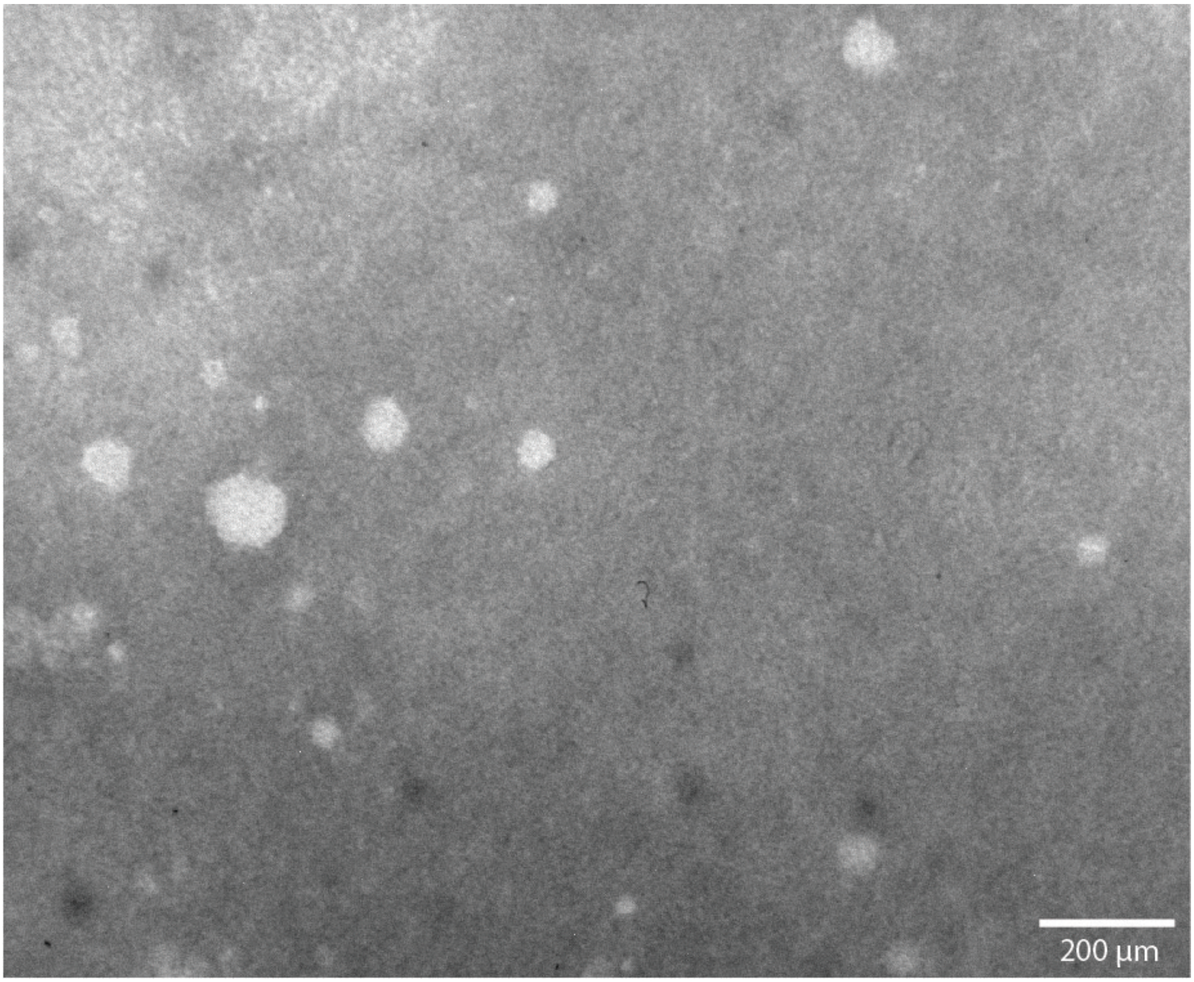

Supplementary Figure 1 – Transmission electron microscopy (TEM) of extracellular vesicles. TEM visualizations were performed using a Zeiss EM10 transmission electron microscope at 60 kV.
